# Detection of Silver Nanoparticles in Seawater Using Surface-Enhanced Raman Scattering

**DOI:** 10.3390/nano11071711

**Published:** 2021-06-29

**Authors:** Monica Quarato, Ivone Pinheiro, Ana Vieira, Begoña Espiña, Laura Rodriguez-Lorenzo

**Affiliations:** International Iberian Nanotechnology Laboratory (INL), Avda Mestre José Veiga, 4715-310 Braga, Portugal; monica.quarato@inl.int (M.Q.); ivone.pinheiro@inl.int (I.P.); ana.vieira@inl.int (A.V.); Begona.Espina@inl.int (B.E.)

**Keywords:** AgNPs, SERS, NPs transformation, water, sensor

## Abstract

Nanomaterials significantly contribute to the development of new solutions to improve consumer products properties. Silver nanoparticles (AgNPs) are one of the most used, and as human exposure to such NPs increases, there is a growing need for analytical methods to identify and quantify nanoparticles present in the environment. Here we designed a detection strategy for AgNPs in seawater using surface-enhanced Raman Scattering (SERS). Three commercial AgNPs coated with polyvinylpyrrolidone (PVP) were used to determine the relative impact of size (PVP-15nmAgNPs and PVP-100nmAgNPs) and aggregation degree (predefined Ag aggregates, PVP-50–80nmAgNPs) on the SERS-based detection method. The study of colloidal stability and dissolution of selected AgNPs into seawater was carried out by dynamic light scattering and UV-vis spectroscopy. We showed that PVP-15nmAgNPs and PVP-100nmAgNPs remained colloidally stable, while PVP-50–80nmAgNPs formed bigger aggregates. We demonstrated that the SERS-based method developed here have the capacity to detect and quantify single and aggregates of AgNPs in seawater. The size had almost no effect on the detection limit (2.15 ± 1.22 mg/L for PVP-15nmAgNPs vs. 1.51 ± 0.71 mg/L for PVP-100nmAgNPs), while aggregation caused an increase of 2.9-fold (6.08 ± 1.21 mg/L). Our results demonstrate the importance of understanding NPs transformation in seawater since this can influence the detection method performance.

## 1. Introduction

Nanomaterials [1] are becoming more and more prevalent as ingredients for several consumer products such as paints, personal care products, food, and cosmetics [2,3,4]. Silver nanoparticles (AgNPs) are becoming, among others, one of the most-used engineered nanomaterials as a result of their properties, mainly their antibacterial properties, in consumer products, including textiles, disinfectants and filtration membranes where the particles can be found in both solid or liquid (coating and spray) state [5]. Despite all the promising applications, there is a growing concern about associated risks to humans and ecosystems. The production, transport, washing, or disposal of products containing AgNPs are only some of the steps that could lead to Ag release into the environment compromising agricultural and fishery activities with a potential impact on human health [6,7,8]. Therefore, there is a growing need for an analytical method to directly detect these NPs present in the environment.

At present, there are different techniques that allow the detection and quantification of AgNPs, including spectroscopic [9] and electrochemical methods [10,11], and, for the majority, single-particle inductively coupled plasma mass spectrometry (sp-ICP-MS) [12,13,14]. Although these techniques allow both qualitative and quantitative determination of NPs, it is crucial to develop equally accurate and fast methods for their characterization and environmental risk assessment on-site. The development of portable sensors for their detection is also necessary since those will allow decentralized monitoring and quick implementation of measures for risk mitigation [15].

Taking advantage of the high scattering and plasmonic properties of AgNPs [16,17], specific detection methods based on surface-enhance Raman scattering can be developed. Unlike Raman spectroscopy, SERS overcomes the limitation due to weak signals by exploiting such AgNPs properties via electromagnetic enhancement mechanism to Raman signal increase [18]. This, coupled with chemical interactions between molecular probe and plasmonic NP and with the ability of metal surfaces to quench the fluorescence background when both are in close proximity, makes SERS sensitive enough to detect trace amounts for analysis even at the single molecule level [19]. A non-overlapped spectra with a narrow bandwidth sensitive to slight changes in molecule structures and orientation is the output of this technique [20]. The applicability of SERS in the detection of nanostructures, like AgNPs, has been already reported, in which a Raman reporter molecule, ferbam (ferric dimethyl-dithiocarbamate), allowed the detection of NPs in complex matrices after strong interactions were established [21].

This work aims to design a detection method for AgNPs by correlating the enhancement of SERS signal of 4-aminobenzenethiol (4ABT)-attached to gold nanostars (AuNSs) with the presence of AgNPs in seawater. Seawater was selected as an environmentally relevant medium because coastal waters are one of the main sinks at the end-of-life of AgNPs-containing products, mainly through treated and untreated wastewater discharges [22]. We selected AuNSs as a highly efficient SERS substrate due to the high localization of the electromagnetic field at their tips and their consequent behavior as individual *hot spots* allowing zeptomole detection of molecules attached to their surface [23,24,25]. 4ABT was chosen as a Raman reporter due to both its aromatic nature that confers a high Raman cross-section and its functional groups, -SH and -NH_2_, which strongly interact with Au and Ag. In fact, 4ABT has been already used as a Raman reporter to detect AgNPs in dietary supplement products by SERS via the formation of S-Ag bond [26]. In this work, we carried out a different approach: First, 4ABT was attached to AuNSs and, subsequently, the free amino group of 4ABT was available bind to AgNPs [27]. Thus, the enhancement of the SERS signal can be explained by inter-particles AgNPs-AuNSs interaction via 4ABT generating *hot spots*, which increases the concentration of electromagnetic field at these sites and, as a consequence, the enhancement of SERS signal also occurs [28]. This enhancement depends on the plasmonic properties of AgNPs, which in turn depend on the physicochemical properties of the NPs such as size, shape, aggregation state, and surface coating. To investigate the effect of these parameters, we selected three commercially available AgNPs coated with polyvinylpyrrolidone (PVP). PVP is a widely used water-soluble polymer whose role as stabilizing agent is well known [29,30]. The molar ratio between silver and macromolecule (i.e., polymer) is usually enough to prevent or at least slow down aggregation and dissolution processes over the time [31]. Having similar surface properties due to the presence of PVP, we studied the effect of the size on SERS enhancement by having two AgNPs with a diameter of 15 (PVP-Ag15nm NPs) and 100 nm (PVP-Ag100nm NPs) and the effect of the aggregation including a predefined product formed by Ag aggregates coated with PVP with a primary NP size of 50–80 nm (PVP-Ag50–80 NPs).

We studied the colloidal stability (i.e., aggregation and dissolution) of selected AgNPs in seawater by UV-vis spectroscopy, dynamic light scattering (DLS), and zeta potential. After understanding the possible transformation of AgNPs in seawater, we performed average SERS detection (i.e., in liquid) in both ultrapure water and seawater to study the possible matrix interference [32] (i.e., salt concentration). In addition, we characterized the AgNPs interaction with 4ABT-coated AuNSs by transmission electron microscopy (TEM) and the colloidal stability of AuNSs in seawater by UV-vis spectroscopy. We were able to construct calibration curves for all AgNPs even the smaller one, PVP-Ag15nm NPs, in seawater. The limits of detection using a portable Raman system, which has lower performance of the detector in comparison with the Raman confocal microscope [33,34], were in the mg/L range.

## 2. Materials and Methods

### 2.1. Silver Nanoparticles (AgNPs)

AgNPs powders with a core diameter of 15 nm (PVP-Ag15nm NPs) and 50–80 nm (PVP-Ag50–80nm NPs) were supplied by SSNano (Houston, TX, USA; product code: 0127SH) and US Research Nanomaterials, Inc (Houston, TX, USA; product code: US1018), respectively, and used without any further purification. The powder composition was 25% wt silver and 75% wt polyvinylpyrrolidone (PVP) for PVP-Ag15nm NPs and 0.2% of PVP for PVP-Ag50–80nm NPs. Further, 1 g/L of AgNPs dispersion was prepared in ultrapure water with a resistivity of 18.2 MΩ at 25 °C (Millipore apparatus, MQ Aquantage A10, Merck, Algés, Portugal) and this was sonicated for 15 min using a bath sonicator (Elmasonic P, Elma, VWR, Amadora, Portugal) (37 kHz, 100% at 25 °C). The dispersion was stored at 4 °C until further use.

An AgNPs ink containing 30% wt AgNPs dispersed in ethylene glycol was purchased from Sigma-Aldrich (Merck Life Science-Sigma Aldrich, Algés, Portugal; product code: 798738). In order to remove ethylene glycol due to its toxicity [35], 4 g of the AgNPs ink were diluted with ultrapure water to reach a concentration of ethylene glycol of 0.15–0.3 M (30–15 mL final volume). The diluted ink was then dialyzed using a 12 kDa cellulose membrane (Merck Life Science Sigma-Aldrich, Algés, Portugal), product code: D6191) against ultrapure water for 6 h. After dialysis, AgNPs were mixed with 24 mL of 150 g/L PVP solution, reaching an Ag:PVP ratio of 1:3 wt. The PVP-Ag100nm NPs dispersion was stored at 4 °C until further use. The purification process and PVP coating of these AgNPs were characterized by dynamic light scattering (SZ-100 device, Horiba, ABX SAS, Amadora, Portugal) and UV-vis spectroscopy (Perkin-Elmer LAMBDA 950 spectrophotometer, Scientific Laboratory Supplies, Wilford, Nottingham, UK). The results are shown in Figure S1.

### 2.2. Synthesis and Functionalization of Gold Nanostars (AuNSs)

The synthesis of AuNSs followed the seed-mediated growth method, in which gold spherical nanoparticles (AuNPs) with a diameter of 13 nm work as seeds for the following star-shaped particles formation.

AuNPs were obtained by in-house synthesis according to the reduction method developed by Turkevich et al. [36] where a small amount of gold salt is reduced by the presence of sodium citrate. Briefly, 250 mL of 0.5 mM of an aqueous solution of HAuCl_4_ (Merck Life Science Sigma-Aldrich, Algés, Portugal) was brought to boil for 5–10 min while being kept under vigorous stirring. Then, 12.5 mL of a warm sodium citrate solution (1% wt/V; Merck Life Science, Sigma-Aldrich, Algés, Portugal) was quickly added. The formation of AuNPs was confirmed by the color change of the dispersion from light yellow to dark red.

Once the synthesis was over, the suspension was cooled down until room temperature was reached and kept in the dark at 4 °C until further use.

For the coating, an aqueous solution containing 530 mg of polyvinilpyrrolidone (PVP) with a molecular weight (MW) of 10K (TCI Europe, Zwinjdrecht, Belgium) was prepared and added to the AuNPs solution to provide a ratio of 60 PVP molecules per nm^2^. The reaction was left overnight under magnetic stirring and the PVP excess was then removed, performing a centrifugation step at 7000× *g* for 90 min. PVP-coated AuNPs were re-dispersed in ethanol and stored in dark condition at 4 °C until further use.

The formation of AuNSs took place by mixing 20 g of PVP-10K with 200 mL of N-dimethylformamide (DMF, Merck Life Science, Sigma-Aldrich, Algés, Portugal) in the presence of 0.5 mM of HAuCl_4_ and 0.023 mM of preformed AuNPs seeds. After a 20 min reaction, the solution became dark blue, and 3 cycles of 60 min centrifugation were performed to finally re-disperse the solution in ethanol.

The functionalization occurred by the conjugation of AuNSs with a Raman reporter molecule, 4-aminobenzenthiol (4-ABT; Merck Life Science, Sigma-Aldrich, Algés, Portugal), using 1:1 molar ratio. If freshly prepared, the reaction took place in 10 min, followed by 3 centrifugation steps in order to remove the molecules excess (3300× *g*, 4 min).

### 2.3. Nanoparticles Characterization

Optical and morphological particles characterization was carried out using UV-vis-NIR spectroscopy (Perkin-Elmer LAMBDA 950 spectrophotometer, Scientific Laboratory Supplies, Wilford, Nottingham, UK), size/zeta potential analyzer (SZ-100 device, Horiba, ABX SAS, Amadora, Portugal), and transmission electron microscopy (JEOL 2100 200 kV TEM, Izasa Scientific, Carnaxide, Portugal). The sample concentration of 12.5 mg/L for PVP-Ag15nm and PVP-Ag100nm and 50 mg/L for PVP-Ag50–80nm was loaded into a quartz cuvette, 10 mm optical path, to perform light scattering and UV-vis analysis. A scattering angle of 90° and a working temperature of 25 °C was used for size determination. For TEM analysis, the particles were subjected to several centrifugation cycles (3 cycles of 60 min at 8960× *g* for PVP-Ag15nm; 3 cycles of 15 min at 2500× *g* for PVP-Ag100nm; and 1 cycle of 10 min at 1500× *g* for PVP-Ag50–80nm) in order to remove the excess PVP that could interfere with the analysis. To prevent particle aggregation, several drops of the suspension were placed on the grid by drying the excess every time. Furthermore, 400 Cu mesh formvar/carbon grids were used for gold investigations, and instead, pure carbon 400 Ti mesh grids were used when silver was analyzed. In both cases, an acceleration voltage of 200 kv was used.

### 2.4. Surface-Enhanced Raman Spectroscopy

For SERS average experiments, 4ABT functionalized AuNSs were chosen as the SERS substrate for the detection of different concentration of AgNPs, in a range 12.5–0.025 mg/L for single particles and 50–0.1 mg/L in the case of aggregates. Different concentrations of AgNPs were added to the AuNSs suspension (1:1, *v*/*v*) and 20 µL of the suspension was then placed on a silicon wafer. The SERS measurements were carried out in the liquid.

The standards were prepared by diluting the testing AgNPs in ultrapure water or synthetic seawater. The artificial seawater was prepared by dissolving a commercial salt (ICA Sal Marinho Basic Plus, Aqualovers, Portugal) in DI water (35 ppm salinity) in order to assess salt interference during detection.

SERS spectra were acquired using a 300 Alpha Confocal Raman (WiTEC, Ulm, Germany) using 10× objective and a portable Raman spectrometer (B&Wtek, ILC-Inst. De Lab. E Cientificos, Lisboa, Portugal) with optical fiber configuration, where 785 nm was the excitation laser line used. The spectra acquisition was performed for 3 s and with 1 scan per measurement and collected using a laser power of 70 mW and 50 mW for the Confocal and the portable Raman, respectively. The resulting SERS spectra were processed using SpectraGriph 1.2.14 software (Software for optical spectroscopy 2016-20 developed by Dr. Friedrich Menges, Oberstdorf, Germany) after being baseline corrected.

## 3. Results

### 3.1. Physicochemical Characterization of AgNPs in Artificial Seawater

**Figure 1:**
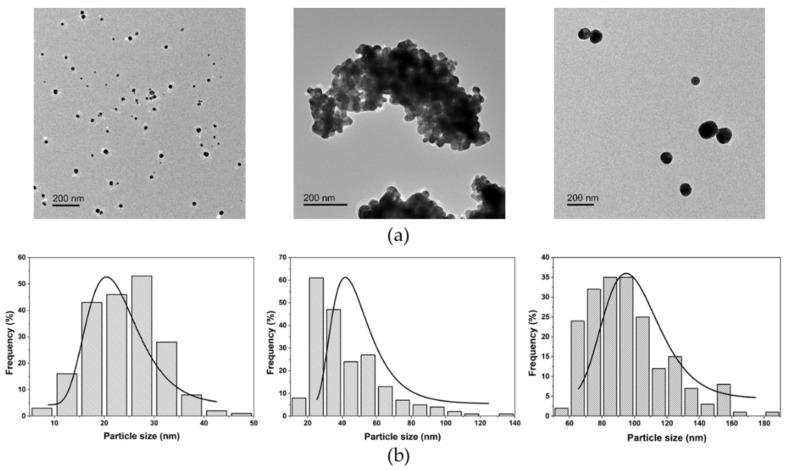
(**a**) TEM images show morphology and particles dispersion. Particles of 15 (PVP-Ag15nm NPs), 50–80 (PVP-Ag50–80nm NPs), and 100 nm (PVP-Ag100nm NPs) are shown. Scale bar of 200 nm. (**b**) Particle size distribution, estimated by measuring an average of 200 particles, and Gaussian fitting are represented by histograms and continuous line, respectively.

We selected three commercial AgNPs coated with polyvinylpyrrolidone (PVP), named here PVP-Ag15nm NPs, PVP-Ag50–80nm NPs, and PVP-Ag100nm NPs. PVP is a well-known stabilizer agent of NPs, and it has been amply used as a stabilizer of AgNPs [37]. Figure 1 shows morphology and size distribution characterized by transmission electron microscopy (TEM). TEM analysis reveals that all NPs present a pseudo-spherical shape and a mean size of 24 ± 7 nm for PVP-Ag15nm NPs, 42 ± 21 nm for PVP-Ag50–80nm NPs, and 96 ± 25 nm for PVP-Ag100nm NPs.

**Figure 2:**
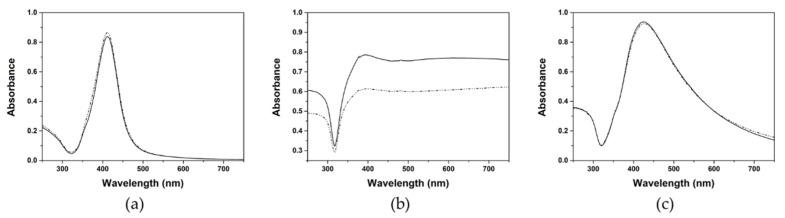
UV-vis extinction spectra of (**a**) PVP-Ag15nm NPs, (**b**) PVP-Ag50–80nm NPs, and (**c**) PVP-Ag100nm NPs dispersed in ultrapure water and artificial seawater, represented by solid line and dotted line spectra, respectively.

AgNPs have unique optical properties resulting in a very particular UV-vis extinction spectrum in the visible range, which corresponds to the typical yellow color. When excited by an electromagnetic field, AgNPs support coherent oscillations of the surface conduction electrons, and this phenomenon, confined oscillations of the charge density, is referred to as localized surface plasmon resonance (LSPR) [38]. This LSPR can be used to obtain information about the colloidal stability of the AgNPs dispersion. Figure 2 shows the UV-vis extinction spectra of three commercial PVP-AgNPs in both ultrapure water and artificial seawater. The extinction spectra of the PVP-Ag15nm NPs and PVP-Ag100nm NPs displayed a single LSPR band centered at 412 nm and 425 nm in both media, which demonstrated that these nanoparticles are colloidally stable in media with high ionic strength like artificial seawater even after 28 days (see Figure S2a–f). This is due to the presence of PVP since this polymer stabilizes the NPs by electrosteric repulsion [39]. However, the extinction spectrum of PVP-Ag50–80nm NPs showed a broad LSPR band developed in the visible-near-infrared region and a decrease of the concentration when in artificial seawater. These features can be attributed to the aggregation of AgNPs, i.e., the plasmonic coupling between closely packed NPs, especially in the presence of high ionic strength. Interestingly, the presence of PVP prevented the collapse running out-of-control and stabilized the formed aggregates in artificial seawater (Figure 2b).

Owing to the fact the polymer shell can act as a dielectric spacer and be an effective insulator, which may hinder additional coupling of LSPR oscillations between associated particles provoking the decrease of the sensitivity of the UV-vis spectroscopy on the aggregation monitoring [40]. DLS overcomes this obstacle and therefore the hydrodynamic size of the AgNPs was also characterized. DLS analysis in ultrapure water confirmed the UV-vis results (Table 1). PVP-Ag15nm NPs and PVP-Ag100nm NPs had a hydrodynamic size of 49 ± 3 nm and 139 ± 2 nm, respectively, which are higher diameters than the diameter obtained by TEM. This is due to the presence of the PVP layer around AgNPs. PVP-Ag50–80nm NPs presented a hydrodynamic size of 619 ± 75 nm confirming the aggregation of these AgNPs. Interestingly, these AgNPs dispersed in ultrapure water presented different NP populations with different sedimentation as shown in the time evolution of the hydrodynamic size in Figure S2h. The presence of PVP is crucial, especially when the particles are dispersed in artificial seawater. Hydrodynamic diameters of 47 ± 2 nm and 97 ± 1 nm are recorded for PVP-Ag15nm NPs and PVP-Ag100nm NPs, showing that the presence of salts does not destabilise the NPs even after 28 days of exposure to this medium (see Figure S2). The value of 1348 ± 407 reported for PVP-Ag50–80nm NPs proves instead that the lower amount of PVP is not enough to prevent aggregation to occur and the particles re-arranged themselves into newly formed aggregates. Moreover, these AgNPs sedimented completely after 1 day of being dispersed in seawater as shown in the time evolution of the UV-Vis extinction spectrum (Figure S2g) and the decrease in the DLS intensity (kcounts) over time (Figure S2j). The hydrodynamic size of PVP-Ag50–80nm NPs displayed an increase in the first 2 h dispersed in seawater and then reached an equilibrium, maintaining a similar aggregate size before complete sedimentation (Figure S2i). The PVP-Ag50–80nm NPs were redispersed after complete sedimentation and their hydrodynamic size were measured by DLS. A similar size was obtained before and after redispersion (Figure S2l).

Zeta-potential measurements reveal a negatively charged surface in the case of AgNPs dispersed in ultrapure water, confirming the repulsion between similarly charged particles in the dispersion (Table 1). These values can be attributed to the negatively charged PVP polymer (zeta potential—30 mV) offering a stabilization of AgNPs due to the combined electrosteric repulsion [39]. A higher negative value of −83 ± 6 mV is recorded for PVP-Ag50–80nm NPs, demonstrating, once again, that the initial aggregates are stable. All AgNPs in seawater showed a decrease (i.e., lower net surface charge) in the zeta potential values: −9 ± 16, −6 ± 10, and −5 ± 10 mV for PVP-Ag15nm NPs, PVP-Ag100nm NPs, and PVP-Ag50–80nm NPs, respectively. This decrease likely is due to the compression of electric double layer (EDL) promoted by the presence of high ionic strength in seawater. This compression of EDL induces aggregation when the only repulse force is electrostatic; however, in this case, the steric repulsions remain due to the presence of PVP on the surface, keeping AgNPs stable. In the case of PVP-Ag50–80nm NPs, an increase of the hydrodynamic size in seawater was observed, which can be attributed to the fact that the concentration of PVP is lower than in the other two NPs (0.2% vs. 75%).

### 3.2. Detection of AgNPs in Artificial Seawater Using SERS

**Figure 3:**
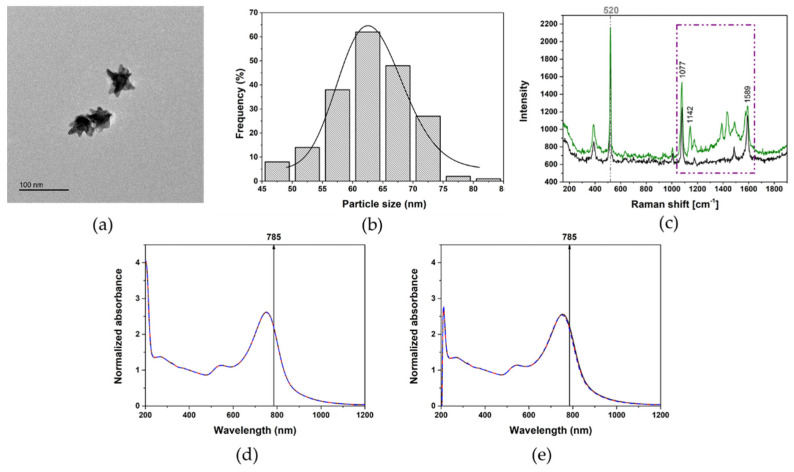
(**a**) TEM image of AuNSs and (**b**) relative particles size distribution and Gaussian fitting estimated on an average of 200 particles analysed. Scale bar of 100 nm. (**c**) Average SERS spectra of 4ABT-functionalised AuNSs (1 mM of AuNSs, black line) and sandwich-like configuration of AgNPs-4ABT-AuNSs (0.5 mM of AuNSs in the presence of 25 mg/L of PVP-Ag100nm NPs, green line). The characteristic peaks of 4ABT (purple dashed rectangle, C-H ring bending 1142 cm^−1^ and ring stretching 1077 and 1589 cm^−1^) are shown. The light-grey dashed line indicates the characteristic peak of silicon (520 cm^−1^) that was used as internal standard. AuNSs stability in (**d**) ultrapure water and (**e**) artificial seawater is shown. Times of 0, 15, and 30 min are represented by black, red, and blue lines, respectively. Black arrows show the wavelength excitation source at 785 nm used in SERS analysis.

We selected AuNSs as the SERS active substrate because they offer an enhancement factor of the Raman signal up to 10^12^ thanks to the lowest-energy, localized surface plasmon mode highly concentrated at the apex of the tips [41,42]. In addition, the core of the AuNSs acts as an electron reservoir due to strong plasmonic coupling effects, contributing significantly to the enhancement of the SERS signal. AuNSs were synthesized (see more details in Methods section) by reducing a gold(III) salt in N,N-dimethylformamide (DMF) in the presence of a high concentration of PVP and preformed 13 nm-spherical PVP-coated Au seeds (see TEM image and UV-vis spectrum in Figure S3). TEM analysis of AuNSs reveals a particle size diameter of 63 ± 7 nm (Figure 3a,b). The UV-vis spectrum of AuNSs showed two LSPR modes, one of them showing a maximum wavelength in the near infrared region (NIR) at 753 nm, related to a plasmon mode localized at the NSs tips and a second lower mode, at 540 nm, associated with the internal core. As a consequence, we selected the 785 nm laser as the excitation source adopted in the SERS measurement because this laser overlaps with the LSPR mode at the tips (see Figure 3d). In addition, this laser was selected to avoid any photodegradation of the probe molecule (4-aminobenzenethiol, 4ABT) used and reduced the possible matrix interference suppressing the fluorescence background and absorption from organic matter dissolved in real water samples.

Before performing SERS experiments, we assessed the colloidal stability of AuNSs in order to confirm that the chosen SERS substrate is not affected by the influence of the surrounding environment. As explained before, level of aggregation, formation of hot-spot regions, and modification of the involved particles could modify SERS sensitivity also affecting system reproducibility. AuNSs were tested for their colloidal stability in both ultrapure water and synthetic seawater for a period of time of 30 min, allowing AgNPs detection. Recorded spectra show how AuNSs are not affected by the surrounding media and the presence of high ionic strength is not able to modify their initial colloidal stability (Data are shown in Figure 3d,e).

SERS is a surface-sensitive technique and, by coupling metallic surfaces with a reporter molecule, it is possible to increase not only the sensitivity but also the specificity toward the interested analyte. We then functionalized AuNSs with 4ABT, which is able to form a self-assembled monolayer (SAM) on the Au surface by forming S-Au bond. As a consequence, a strong SERS signal in suspension (i.e., average SERS) was recorded (Figure 3c, black spectrum) [43].

**Figure 4:**
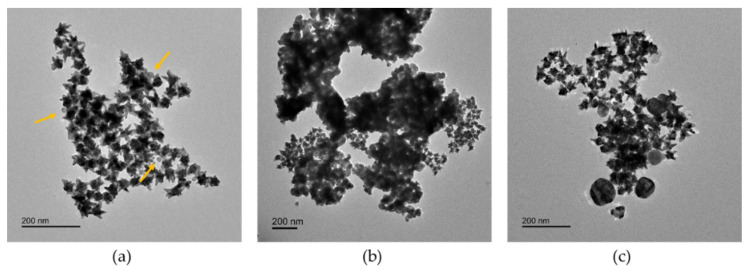
TEM images of AgNPs-4ABT-AuNSs interaction. (**a**) PVP-Ag15nm NPs, (**b**) PVP-Ag50–80nm NPs, and (**c**) PVP-Ag100nm NPs in ultrapure water. Scale bar of 200 nm. Yellow arrows point AgNPs.

This average SERS spectrum displays three peaks at 1589, 1077, and 390 cm^−1^, which correspond to ring stretching vibrations. The amine group of 4ABT, in para-substitution, has a strong interaction with Ag surface, allowing a specific detection of AgNPs. This interaction between the amine group and AgNPs generated very clear changes in SERS spectrum of 4ABT (Figure 3c, green spectrum). Apart from the obvious enhancement of the SERS intensity, four additional peaks are observed: 1142, 1175, 1390, and 1431 cm^−1^ that correspond to C-H ring bending vibrations. Despite its extensive use in SERS, it is common to consider *p*-ABT as a molecule with an abnormal enhancement mechanism depending on both substrates (i.e., Au and Ag) and used experimental conditions [26,27]. In this case, our system could be considered, which forms a sandwich-like configuration of AuNSs-4ABT-AgNPs (see Figure 4a) and the selective enhancement of those peaks can be associated with the charge transfer mechanism (i.e., charge transfer between both metal NPs coupled with the vibrations of 4ABT), as reported by Zhou et al. [43].

**Figure 5:**
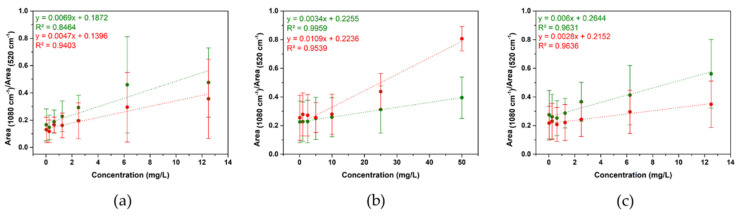
Calibration curve of (**a**) PVP-15nmAg NPs, (**b**) PVP-50–80nmAg NPs, and (**c**) PVP-100nmAg NPs detection in ultrapure water (green line) and artificial seawater (red line).

In this work, we proposed an indirect detection based on changes in the 4ABT SERS signal in presence of AgNPs. We chose this indirect SERS strategy because it increases the selectivity by covalent interaction of the amino group and Ag surface and the enhancement of SERS signal produced by the formation of sandwich-like configuration AgNPs-4ABT-AuNSs can be correlated with the AgNPs concentration. The correlation among silver concentrations was determined by the analysis of the band at ~1080 cm^−1^, the one supposed to arise from electromagnetic field enhancement coupled with chemical enhancement [44]. The in-suspension SERS analysis was performed (i.e., the measurements were done in liquid) and all integrated areas under the 1080 cm^−1^ peak were normalized with respect to those of silicon wafers (520 cm^−1^) used for support and internal standard. For an on-field monitoring application, this average SERS strategy should be implemented into a portable system. Thus, average SERS spectra of 4ABT on AuNSs in the presence of different concentration of PVP-15nmAg NPs with a portable Raman system and a confocal Raman system with a 10× objective were acquired to compare the performance. The SERS measurements were carried out both in ultrapure water, as a control medium, and artificial seawater. Figure 5a and Figure S4c show the calibration curve, where the error bars indicate standard deviations from three independent experiments, obtained in both media (ultrapure water, in green, and artificial seawater, in red) and using the two Raman systems. For both ultrapure water and seawater, the plot of the ratio between the areas under the peaks at 1080 and 520 cm^−1^ against AgNPs concentration showed a good linear correlation with r^2^ values of 0.8464 (ultrapure water) and 0.9403 (seawater) for portable Raman system and 0.9162 and 0.9137 for confocal Raman system, when PVP-Ag15nm NPs were analyzed. The limit of detection (LoD) was calculated from the sensitivity of the calibration curve using the equation 3.3 *SDy*/*a*, where *SDy* is the standard deviation of the response of the curve and *a* is the slope of the calibration curve. LoD values were similar for both the media and the two Raman systems: 3.08 ± 1.47 (ultrapure water) and 2.15 ± 1.22 mg/L (seawater) for the portable Raman system and 3.12 ± 2.28 (ultrapure water) and 2.17 ± 0.93 mg/L for the confocal Raman system. The similar performance shown for both systems can be explained by the fact that we selected an average SERS strategy (i.e., in liquid) for AgNPs detection, and therefore we did not take advantage of the better spatial resolution of the confocal Raman system.

We performed SERS analysis about the possible effect of the particle size and aggregation degree of AgNPs using only the portable Raman system. Thus, we carried out the set of SERS measurements using the same conditions as before for PVP-15nmAg NPs, PVP-100nmAg NPs, and PVP-50–80nmAg NPs. Figure 5 shows the calibration curves for PVP-50–80nmAg NPs (b) and PVP-100nmAg NPs (c) obtained in both ultrapure water and artificial seawater. A better fit was obtained related to PVP-100nmAg NPs (r^2^ of 0.9631 in ultrapure water and 0.9636 in seawater) and PVP-50–80nmAg NPs (r^2^ of 0.9959 in ultrapure water and 0.9539 in seawater). This is probably due to the better coupling of the LSPR band (see Figure 2b,c) to the 785 nm excitation wavelength and the higher local electromagnetic field generated from NPs with bigger size [45]. However, similar LoD values were obtained in ultrapure water: 2.28 ± 1.46 mg/L for PVP-100nmAg NPs and 3.75 ± 1.00 mg/L for PVP-50–80nmAg NPs, which may be related to AgNPs-4ABT-AuNSs sandwich-like configuration (see Figure 4b,c). The enhancement is produced in the gap between AuNSs and AgNPs and non-all surface area of AgNPs is covered by 4ABT. Interestingly, we observed different behavior of the detection in seawater. The LoD values were similar for AgNPs that remained colloidally stable in seawater (see Figure 2, Table 1 and Table S2), i.e., PVP-15nmAg NPs (LoD = 2.15 ± 1.22 mg/L) and PVP-100nmAg NPs 1.51 ± 0.71 mg/L), while LoD for Ag aggregates increased 4-fold (6.08 ± 1.21 mg/L), demonstrating that aggregation has an extremely high impact on the sensitivity of our average SERS approach. This can be explained in terms of decreasing the available surface area that interacts with 4ABT-AuNSs (see Figure 4b). Nonetheless, the integration of a pre-concentration step will be a considerable improvement to bring the detection level down to an order of magnitude relevant to environmental concentrations [46].

## 4. Discussion

Herein, we have designed an average SERS (i.e., in-suspension SERS) strategy for the detection of PVP-coated AgNPs in artificial seawater using a portable Raman system. We selected 4ABT as a chemoreceptor to trap the AgNPs, which was already used as an SERS reporter for AgNPs detection in dietary supplement products and nasal spray [47]. However, higher AgNPs concentrations were used (20 mg/L) and a microRaman spectrometer was used for detection. In this study, the main aim was to design a detection system that could be implemented in a portable Raman system, allowing its future use for point-of-care monitoring, despite the initial limitations of those systems such as less spatial resolution, less sensitivity, less reproducibility, and lower control of emission losses. To this aim, we performed the measurement in a suspension [48] and used a silicon wafer as an internal standard, which helped in overcoming those limitations. This, together with the short time for analysis and being a non-destructive technique, makes SERS a promising approach for the detection of AgNPs traces in the aquatic environment, regardless of size and particles aggregation. With this in-suspension SERS strategy, it was possible to detect a concentration of AgNPs down to 1.51 ± 0.71 mg/L. The sensitivity was affected by the aggregation of AgNPs, increasing to almost 4-fold in the LoD when NPs were aggregated.

It is important to note that despite the high demand for portable systems that can detect and characterize these new emerging nanocontaminants, the innovation of these portable systems has been focused on the detection of nanoplastics [15,49] and pathogens at nanoscale (e.g., virus) [50]. This could be because of the wide variety of plastics found in the ocean at a high concentration: 11.6–21.1 million tons in the Atlantic Ocean [51]. However, the effort of the development of these portable systems should also address the detection of engineered inorganic nanoparticles (EINPs) in the aquatic system. The predicted environmental concentration (PEC) for these EINPs covers a wide range from the highest PEC for titanium dioxide NPs in the scale of mg/L [22] to a concentration of ng/L of total silver for AgNPs [46]. Taking into account the PEC for AgNPs to be able to implement our average SERS methods, a pre-concentration step must be integrated. The utilization of solid phase extraction [52] or cloud point extraction [53] has been reported for this purpose; however, these were coupled to “non-portable” analytical methods: Flame atomic absorption spectrometry and inductively coupled plasma mass spectrometry, respectively.

Despite the appeal of this strategy, important challenges hinder the implementation of the portable sensors based on SERS: (1) The transformation of these nanocontaminants as a function of chemical transformation (e.g., aggregation or dissolution and (2) the matrix interferences (e.g., sulfidation), organic matter or the presence of others plasmonic NPs), which compromise the robustness, selectivity, and sensitivity of the SERS-based method. In the case of the effect of AgNPs transformation, we already demonstrated the negative impact of aggregation on the SERS strategy increasing the LoD4-fold. The dissolution could apparently have less impact since the variation of the sensitivity of the method as a function of the size was minimal (PVP-15nmAg NPs LoD 2.15 ± 1.22 mg/L and PVP-100nmAg NPs LoD 1.51 ± 0.71 mg/L). The formation of Ag_2_S from the sulfidation of AgNPs in seawater [54] would have an important impact on the SERS enhancement, which does not mean “a decrease in the signal”. The contributions of charge-transfer in the SERS effect of Ag and Ag_2_S NPs are different, being higher in the case of Ag_2_S as reported by Fu et al. [55]. Moreover, other noble metal NPs that could be released into the environment at significant concentrations would be platinum, palladium, or copper NPs from the catalysis industry and the wood preservation industry. Therefore, we cannot rule out their interference in the detection system and the calibration curves for AgNPs detection must be performed in the presence of these NPs (including Ag_2_S). It is then possible that mixed signals will be obtained with the added contribution of several types of plasmonic NPs preventing the exact identification; however, in this case, we may be able to extract a correlation in terms of “AgNPs equivalents”, which would nevertheless have high impact as an early warning system.

Given the promising results, the next steps will be to develop a portable device involving a sample preparation module coupled with the SERS-based detection module in order to reduce the challenges presented by on real samples, reduce the lack of specificity and noise, and increase the robustness of these sensors.

## Figures and Tables

**Table 1:** Physicochemical characterization of selected AgNPs.

	Ultrapure Water			Artificial Seawater		
	PVP-15nmAg NPs	PVP-100nmAg NPs	PVP-50–80nmAg NPs	PVP-15nmAg NPs	PVP-100nmAg NPs	PVP-50–80nmAg NPs
**Hydrodynamic diameter ^1^ (nm)**	49 ± 3	139 ± 2	618 ± 83	47 ± 2	97 ± 1	1348 ± 407
**PDI ^2^ (%)**	57 ± 7	20 ± 2	146 ± 11	36 ± 3	27 ± 4	78 ± 12
**Z potential ^3^ (mV)**	−24 ± 5	−21 ± 12	−83 ± 6	−9 ± 16	−6 ± 10	−5 ± 10

^1^ Mean hydrodynamic diameter and polydispersity index. ^2^ obtained by DLS at a scattering angle of 90° and 25 °C. DLS measurements were carried out by quintupled mean ± standard deviation (SD). ^3^ Zeta potentials were measured in 5 runs (mean ± SD).

## Data Availability

The data presented in this study are available on request from the corresponding author. The data are not publicly available due to the database is still developing and the public access is still limited.

## Supplementary Materials

The following are available online at https://www.mdpi.com/article/10.3390/nano11071711/s1, Figure S1: Characterization of physicochemical properties of AgNPs with a diameter of 100 nm as a function of each purification and functionalization step; Figure S2: Spectral evolution of optical absorbance and hydrodynamic size evolution of PVP-15nmAg NPs (upper line) and PVP-100nmAg NPs; Figure S3: Physicochemical characterization of spherical AuNPs with a diameter of 15 nm; and Figure S4: SERS analysis of PVP-15nmAgNPs dispersed in both ultrapure water and artificial seawater using a confocal Raman microscope.
