# Supplementary material for: Detection of Silver Nanoparticles in Seawater Using Surface-Enhanced Raman Scattering
Source: Nanomaterials (Basel). 2021 Jun 29;11(7):1711. doi: 10.3390/nano11071711 (PMC8308189; doi:10.3390/nano11071711)
Supplement: Supplementary file 1 [file nanomaterials-11-01711-s001.zip › nanomaterials-1266150-supplementary.pdf]

nanomaterials

Supporting information

Detection of Silver Nanoparticles in Seawater Using Surface-Enhanced Raman Scattering

Monica Quarato[1], Ivone Pinheiro[1], Ana Vieira[1], Begoña Espiña[1], Laura Rodriguez-Lorenzo[1*]

[1] International Iberian Nanotechnology Laboratory (INL), Avda Mestre José Veiga, 4715-310, Braga, Portugal; monica.quarato@inl.int (M.Q.); ivone.pinheiro@inl.int (I. P.), ana.viera@inl.int (A. V.); Begona.Espina@inl.int (B. E.); laura.rodriguez-lorenzo@inl.int (L.R.-L.)

* Correspondence: laura.rodriguez-lorenzo@inl.int (L.R.-L.)

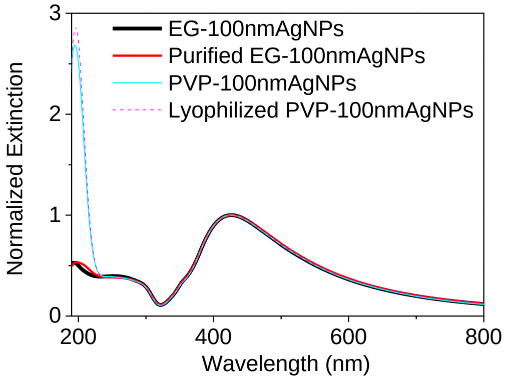

NPs	[NPs] / mg/L	D_H[a] (PDI)[b] / nm
		Ultrapure water
**EG[C]-100nmAgNPs**	13.3	120 ± 1 (0.19 ± 0.06)
**Purified EG-100nmAgNPs**	60	110 ± 2 (0.28 ± 0.04)
**PVP-100nmAgNPs**	60	120 ± 1 (0.29 ± 0.04)
**Lyophilazed PVP-100nmAgNPs**	60	131 ± 1 (0.25 ± 0.04)

[a] Mean hydrodynamic diameter was obtained by DLS at room temperature and at a scattering angle of 90° for 60 s. DLS measurements were carried out on 5 runs: Mean ± Standard desviation (SD).
[b] Polydispersity index
[c] EC=Ethylene glycol

Figure S1. Characterization of physico-chemical properties of AgNPs with a diameter of 100 nm as a function of each purification and functionalization step. AgNPs remained colloidally stable after purification and PVP functionalization (PVP-100nmAgNPs).

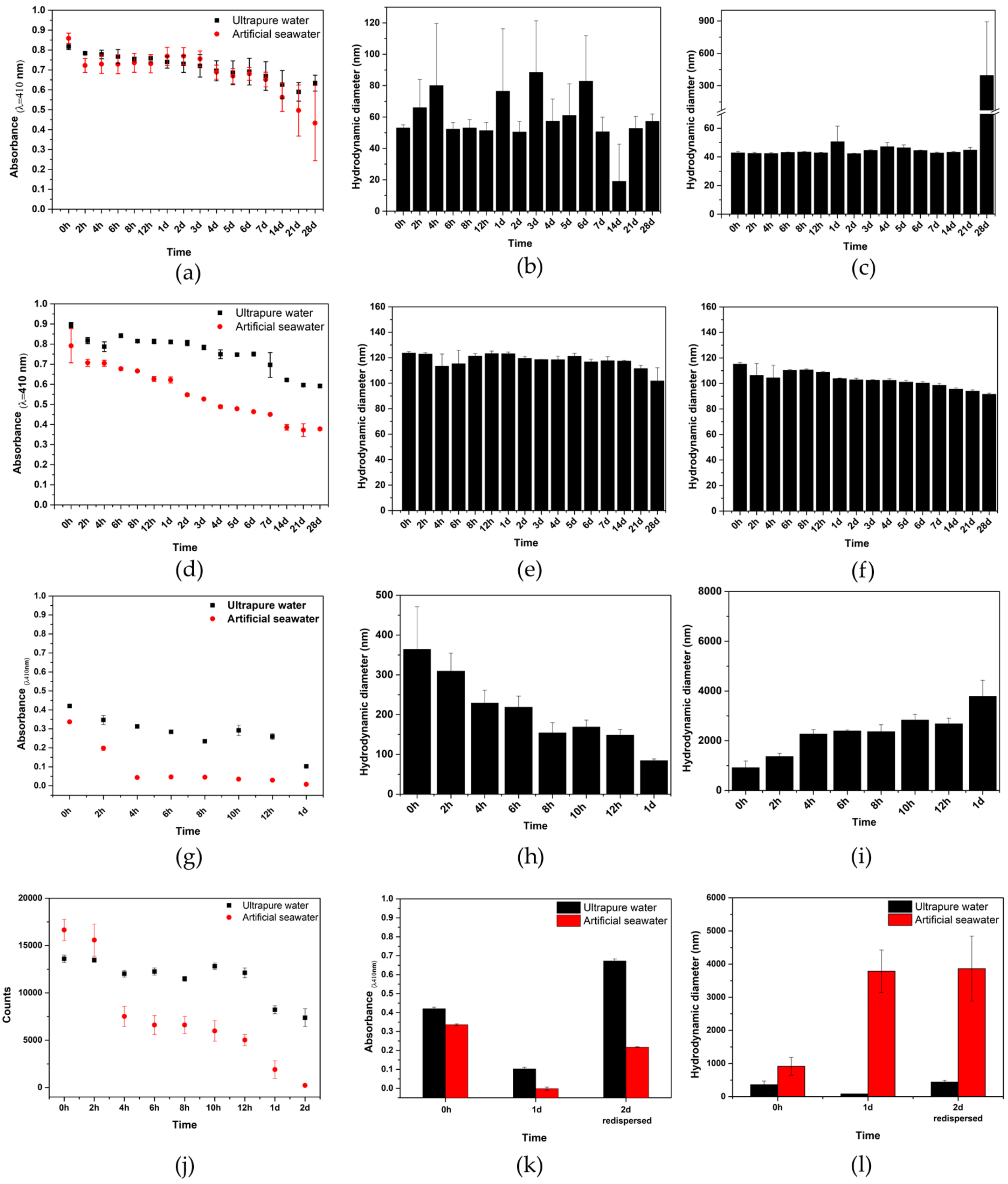

Figure S2. (a, d, g) Spectral evolution of optical absorbance and hydrodynamic size evolution (obtained by DLS, scattering angle of 173° and 20 °C) of PVP-Ag15nm NPs (first line), PVP-Ag100nm NPs (second line), PVP-Ag50-80nm NPs (third line). The NPs are dispersed in both (b, e, h) ultrapure water and (c, f, i) artificial seawater at initial concentration of 12.5 mg/L for PVP-Ag15nm NPs and PVP-Ag100nm NPs and 50 mg/L for PVP-Ag50-80nm NPs. The particles were monitored over 30 days for single particles and 1 day for the aggregates at room temperature. In the case of PVP-Ag15nm NPs, the particles stay colloidally stable over the time. In the case of PVP-100nmAg NPs, a decay of LSPR band is observed over time, which is due to likely the sedimentation of the NPs. In the last case, PVP-Ag50-80nm NPs show not only a decay of LSPR band due to the fast sedimentation but also a variation in the hydrodynamic size where the sedimentation of big aggregates occurs since the first hours. (j, k) Particles counts of PVP-Ag50-80nm NPs demonstrating that after 1 day, complete sedimentation occurs. This is also confirmed by the decay of LSPR band at 1 day and its consequently increase after redispersion. (l) Hydrodynamic size evolution of PVP-Ag50-80nm NPs when in ultrapure water and artificial seawater revealing that despite the fast sedimentation, the particles keep the same size after redispersion.

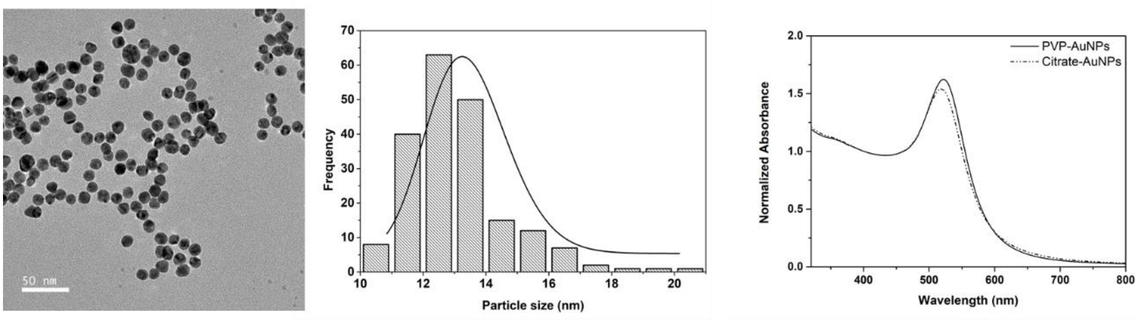

(a) (b) (c)

Figure S3. (a) Representative TEM images of spherical AuNPs used as seed in the AuNSs synthesis and (b) their histogram. The TEM analysis relevels a diameter of 13 ± 2 nm. (c) UV-Vis spectrum before and after PVP coating of AuNPs.

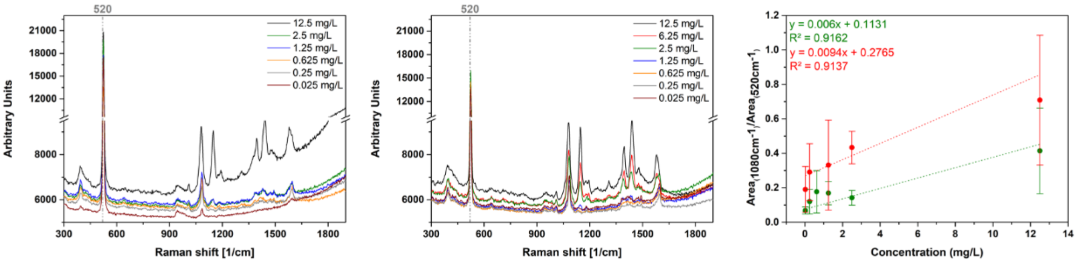

(a) (b) (c)

Figure S4. SERS analysis of PVP-15nmAgNPs dispersed in both (a) ultrapure water and (b) artificial seawater at different concentration. A confocal Raman microscope was used to perform the average SERS experiments using a 10× objective and 785 nm as excitation laser line. (c) Experimentally determined calibration curves for the detection of these AgNPs in ultrapure water (green) and artificial seawater (red). A linear relationship between the AgNPs concentration and the normalized area under the peak at 1080 cm^{-1} was found at the range study here. The light-grey dashed line indicate the characteristic peak of silicon (520 cm^{-1}).
